# Two Oral Midazolam Preparations in Pediatric Dental Patients: A Prospective Randomised Clinical Trial

**DOI:** 10.1155/2015/349795

**Published:** 2015-05-20

**Authors:** Katayoun Salem, Shaqayegh Kamranzadeh, Maryam Kousha, Shahnaz Shaeghi, Fatemeh AbdollahGorgi

**Affiliations:** ^1^Department of Pediatric Dentistry and Oro-Maxillofacial Developmental Disease Research Center, Faculty of Dentistry, Guilan University of Medical Sciences, Rasht 4194173774, Iran; ^2^Guilan University of Medical Sciences, Rasht, Iran; ^3^Department of Pediatric Psychiatry, Shafa Hospital, Guilan University of Medical Sciences, Rasht, Iran; ^4^Department of Pediatric Anesthesiology, Mofid Hospital, Shahid Beheshti University of Medical Sciences, Tehran, Iran; ^5^Medical Research Development Center, Mofid Children's Hospital, Shahid Beheshti University of Medical Sciences, Iran

## Abstract

Pharmacological sedation is an alternative behavior management strategy in pediatric dentistry. The aim of this study was to compare the behavioral and physiologic effects of “commercially midazolam syrup” versus “orally administered IV midazolam dosage form (extemporaneous midazolam (EF))” in uncooperative pediatric dental patients. Eighty-eight children between 4 to 7 years of age received 0.2–0.5 mg/kg midazolam in this parallel trial. Physiologic parameters were recorded at baseline and every 15 minutes. Behavior assessment was conducted objectively by Houpt scale throughout the sedation and North Carolina at baseline and during injection and cavity preparation. No significant difference in behavior was noted by Houpt or North Carolina scale. Acceptable behavior (excellent, very good, and good) was observed in 90.9% of syrup and 79.5% of EF subjects, respectively. Physiological parameters remained in normal range without significant difference between groups and no adverse effect was observed. It is concluded that EF midazolam preparation can be used as an acceptable alternative to midazolam syrup.

## 1. Introduction

Dental fear/anxiety and behavior management problems in pediatric patients are two major aspects of uncooperativeness in pediatric dentistry. Whilst Psychological techniques are the cornerstone of behavior management in pediatric dentistry, a number of children cannot cope with dental treatment using these techniques alone. Pharmacological methods have been used as an adjunct to enhance child cooperativeness and facilitate dental treatment [1, 2]. The primary aim of mild and moderate pharmacological sedation in pediatric dentistry is to modify the patient's behavior to an extent that allows applying behavior management techniques [1]. Evidence for best choice of sedative agents in children is still incomplete [2]; however, midazolam, a short-acting benzodiazepine, has been extensively used due to its therapeutic index and wide margin of safety [3–9]. Since oral dosage form of midazolam is not commercially available in many countries, therefore injectable midazolam in mixture with a flavoring agent, to mask the undesirable taste and adjust the pH, briefly known as extemporaneous form (EF) has been used as an alternative [3, 7].

Despite extensive administration of EF preparation, there is no consensus on its effectiveness [2]. Therefore the aim of this study was to

- evaluate and compare the safety and efficacy of two midazolam oral preparations: “EF midazolam” versus “commercially prepared midazolam syrup” in pediatric dental patients by measuring physiological parameters and behavioral scales including* North Carolina* and* Houpt behavioral scales*. The latter ones evaluate the level of consciousness and cooperativeness in sedated patients.

## 2. Subjects and Methods

The research protocol was reviewed and approved by the Ethical Committee of the Guilan University of Medical Sciences, Rasht, Iran (Grant number 3910354611). The investigation was a randomized, double-blind study. Written informed consent was obtained from parents or legal guardians prior to enrollment of subjects in the study.

The study was conducted in 88 children (41 males and 47 females) aged 4 to 7 years and ASA class I (ASA: American Society of Anesthesiologists' Physical Status Classification System) that means a normal healthy subject. The patients were referred to Pediatric Clinic of Guilan Dental School due to uncooperativeness and difficulties in behavior management. Prior to sedation appointment, it was attempted to treat these children using behavior management methods including Tell-Show-Do and positive reinforcement. Eligible participants were rated as negative on the Frankl behavioral rating scale [10]. Children were required to have an adequate degree of understanding to communicate during the course of treatment. With regard to dental treatment, participants must have at least one primary molar with pulp treatment needs. Patients were excluded from the study if they had physical or mental disabilities, history of respiratory diseases in the past two weeks, and tonsil/adenoid hypertrophy that occupied more than 50% of pharyngeal space. Other exclusion criteria included anatomical deformities in face and neck such as micrognathia and macroglossia and any known allergy to midazolam.

Eligible patients were randomly assigned to one of two parallel treatment groups. Forty- four patients were randomly allocated to each group using 6 quadric blocks. Patients received 0.2 or 0.5 mg/kg midazolam syrup or EF midazolam according to their age up to a maximum dose of 12 mg. A dose of 0.5 mg/kg was administered to children less than five years of age (48 to 59 months) and 0.2 mg/kg for over the age of five years (60 to 84 months) (Saarnivaara 1998). Demographic information of the study patients is summarized in Table 1.  Table 2 presents the age-adjusted distribution of children.

Sample size was calculated based on a previous sedation study [8] with 80% study power and 95% confidence interval (*α* = 0.05) and the clinical difference between groups was considered 25%.

### 2.1. Drug Administration

Children received either regimen A: 0.2 or 0.5 mg/kg midazolam maleate syrup sugar free liquid (Amsed oral liquid 2.5 mg/mL; UK) or regimen B: 0.2 or 0.5 mg/kg midazolam EF 2.5 mg/mL that consisted of the injectable solution of midazolam hydrochloride (Ampule 15 mg/3 mL Midamax, Tehran Shimi, Iran) in combination with the same volume of concentrated viscous orange syrup according to their age. The pH of final EF preparation was 3.7 and the commercial preparation's pH was equal to 4. Both pH values were determined by automated pH meter (Acorn Ion 6 Meter). Drugs were administrated by a dental nurse who was unaware of the study design, using a needleless syringe placed in the lower buccal vestibule. After administration of midazolam, the patients remained under supervision in a quiet place. Thirty minutes after administration, children were transferred to dental operatory in accompany of their parents. Parents were asked to leave the operatory during the treatment session to eliminate any potential impact of parent's presence on child's behavior. All participants received similar restorative treatments including topical anesthetic gel (Benzocaine 10%) followed by local anesthesia (Lidocaine-Epinephrine 1/100.000) and pulp therapy followed by restoration of the tooth which included either fillings or stainless steel crowns. The maximum dose of local anesthesia was limited to 4 mg/kg. Treatment was planned to be accomplished in maximum thirty minutes and any type of physical restraints was not used.

### 2.2. Behavior Assessment

Behavior was videotaped and rated by a trained and calibrated examiner who was blind to the study design using two behavioral measures: North Carolina rating scale to evaluate behavior preoperatively and during critical moments of treatment including injection and cavity preparation (Table 3) and Houpt sedation rating scale to determine the level of sedation according to degree of sleep, crying, movement, and finally an overall appraisal of behavior through the treatment course (see Definition of Houpt sedation rating scale) [11]. The examiner was the senior dental student who was trained and calibrated via examination of five patients prior to study. The Cohen Kappa for intra-examiner reliability was 8.9.

*Definition of Houpt Sedation Rating Scale Is as Follows [11]*

*Rating Scale for Sleep*

1. fully awake, alert,
2. drowsy, disorientated,
3. asleep.

*Rating Scale for Crying*

1. hysterical crying that demands attention,
2. continuous, persistent crying that makes treatment difficult,
3. intermittent, mild crying that does not interfere with treatment,
4. no crying.

*Rating Scale for Movement*

1. violent movement interrupting treatment,
2. continuous movement making treatment difficult,
3. controllable movement that does not interfere with treatment,
4. no movement.

*Rating Scale for Overall Behavior*

1. aborted no treatment rendered,
2. poor treatment interrupted, only partial treatment completed,
3. fair treatment interrupted, but eventually all completed,
4. good, difficult, but all treatment performed,
5. very good some, limited crying or movement, for example during anesthesia or mouth prop insertion,
6. excellent no crying or movement.

### 2.3. Physiologic Parameters

Physiologic parameters including heart rate, respiratory rate, oxygen saturation, and blood pressure were monitored continuously during treatment using operating room design monitors (Massimo Set, Alborz B5 Saadat, Iran) through the course of treatment and every 15 minutes. Physiological parameters were recorded at baseline and at every 15 minutes. The parameters rated at the time of arrival when the child entered the operatory on the day of study for sedation appointment and before any drug administration. The measurements repeated at the beginning of treatment and then every 15 minutes. Hypoxemia was determined as oxygen saturation beyond 93% (except during crying) and Spo2 < 90% was considered as apnea [12].

### 2.4. Data Analysis

Data were analyzed using Kolmogorov Simonov, independent *t*-test, Chi-square, Friedman test, Fisher's exact test, Mann-Whitney test, repeated measure Greenhouse Geiseer, adjustment Bonferroni, backward stepwise multivariate regression, and Mantel-Haenszel common odds ratio by SPSS version 21. Significance level was established at 0.05 in all tests.

## 3. Results

A total of eighty-eight children including participated in the study. Demographic characteristics of patients are presented in Table 1. There were no statistical differences in age, weight (Independent *t*-test *P* = 0.14, and *P* = 0.77), and gender (Chi-square *P* = 0.41) between the two groups. Characteristics of participants under and above the age of 60 months in two study groups are presented in Table 2. Participants of syrup and EF groups did not show significant difference in age and gender.

**Figure 1:**
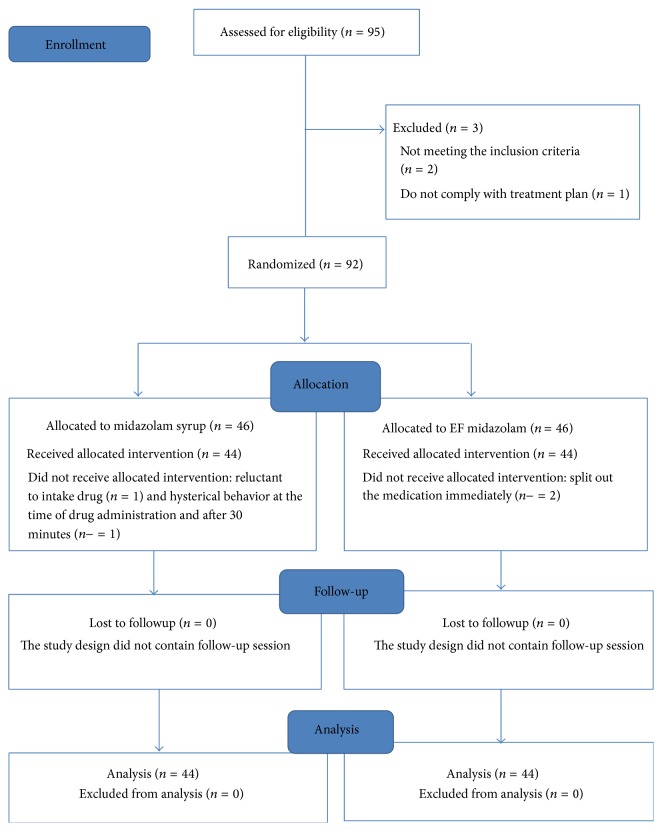
Consort flow diagram of study group.

The flow diagram of study participants is presented in Figure 1.

### 3.1. Rating of Behavior 

#### 3.1.1. North Carolina Rating Scale

Table 2 presents the behavior assessments by North Carolina rating scale at important moments of sedation appointments including “before sedation, when the child arrived to operatory at the day of sedation appointment and before any drug administration,” “during injection,” and “cavity preparation.” The worst manifestation of child's behavior was recorded at each step. All children revealed more calm behavior at the times of injection and cavity preparation in comparison to their behavior at the time of arrival (Friedman test *P* < 0.001).

#### 3.1.2. Houpt Sedation Rating Scale

**Figure 2:**
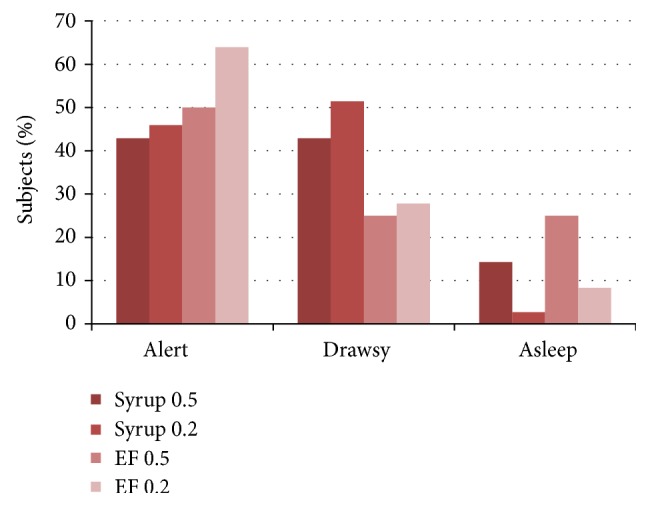
Percentage of children in Houpt categories of sleep in two study groups adjusted for age (dose 0.5 or 0.2 mg/kg).

**Figure 3 fig3:**
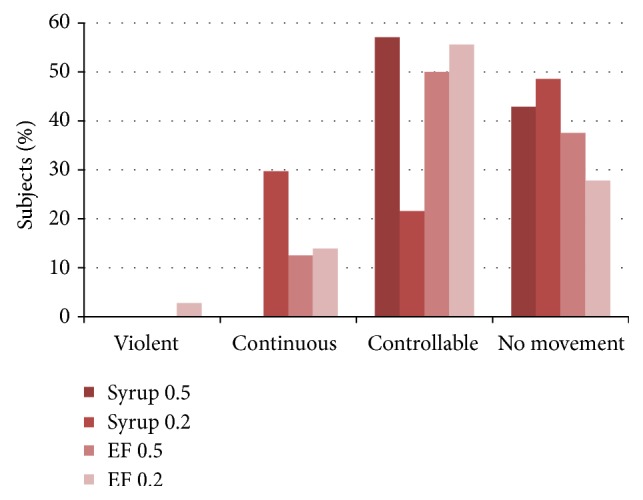
Percentage of children in each of Houpt categories of movement in two study groups adjusted for age (dose 0.5 or 0.2 mg/kg).

**Figure 4 fig4:**
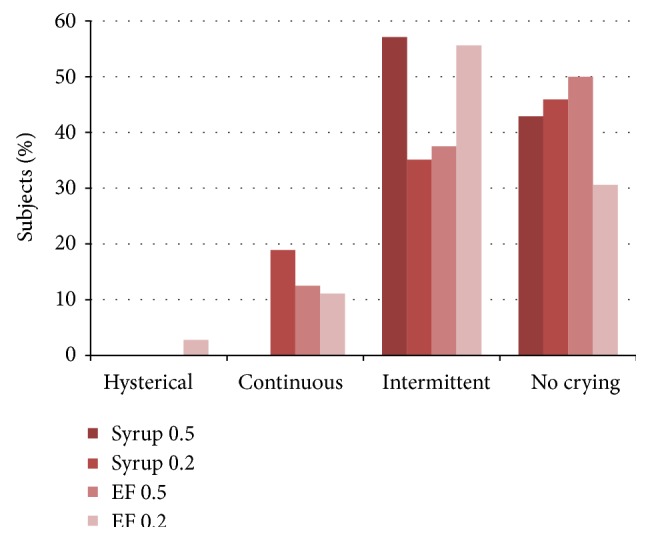
Percentage of subjects in each of Houpt categories of crying in two study groups adjusted for dose/age (dose 0.5 or 0.2 mg/kg).

**Figure 5:**
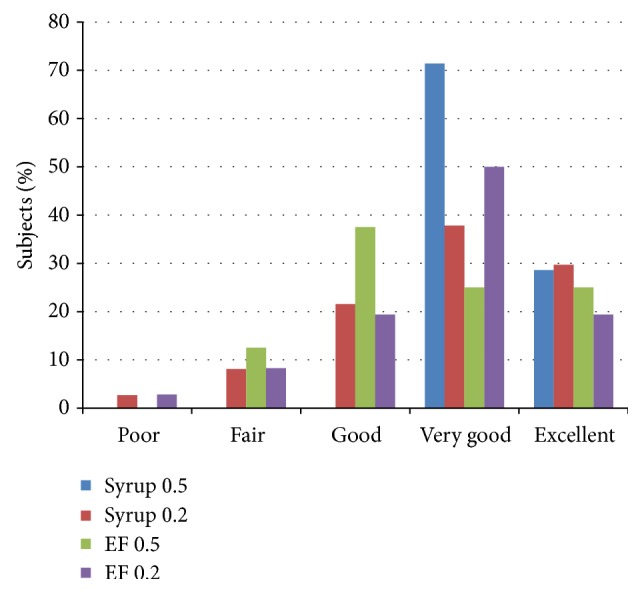
Percentage of subjects in each of Houpt categories of overall behavior in two study groups adjusted for age/dose (dose 0.5 or 0.2 mg/kg).

Figures 2 [Fig fig3] [Fig fig4]–5 present the age adjusted categories of* Houpt Behavior Rating Scale*. There were 15 children under the age of 59 months and 73 aged 60 months and older. Ratings of sleep, crying, movement, and overall behavior domains were divided into two-dose (age) groups that did not differ significantly: Mann-Whitney test; *P* = 0.29, *P* = 0.41, *P* = 0.45, *P* = 0.33, respectively (Figures 2–5).

Acceptable behavior according to age was observed in 7 and 6 children younger than the age of 5 years and among 33 and 29 children at 5 years of age and older, in syrup and EF groups, respectively (Fisher exact; *P* = 0.267 and *P* = 0.261).

When adjusted for age (48–59 months children versus 60 months and older), the risk that a child displayed acceptable overall behavior after EF midazolam administration was 0.38 times compared to those who received syrup (OR = 0.388, SE (0.64) 95% CI: 0.11–1.37; *P* = 0.114) (Mantel-Haenszel common odds ratio estimate).

The overall behavior regardless of age was dichotomized, and two categories of acceptable and unacceptable behavior were determined. The Acceptable behavior was defined as* good, very good, and excellent.* The unacceptable behavior included:* fair, poor, and aborted* categories of behavior. Acceptable behavior was observed in 90.9% of syrup and 79.5% of EF, respectively, Chi-square *P* = 0.13.

Stepwise backward regression was used to determine the best predictors of overall sedation success regardless of age dichotomization. Final model showed that the only predictor of sedation success was administration of commercially prepared syrup that increased the chance of success by 4.67 times: *P* = 0.04; OR: 4.67 (95% CI: 1.05–20.6).

The palatability of drugs was rated as acceptable by 79.5% of those where syrup was administered and 59% of EF group children (Chi-square *P* = 0.06).

### 3.2. Physiologic Parameters

Data on physiological parameters are presented regardless of age classification. Investigated physiologic parameters remained in normal range in both study groups. Up to 20% increase/decrease in normal limits for each age group was considered normal. A detailed explanation of physiological parameters is presented in the following.

#### 3.2.1. Percent of Oxygen Saturation (SPO_2_)

There was no significant difference between two study groups in percent of oxygen saturation: *P* = 0.24 (repeated measure Greenhouse Geiseer). Fluctuation of SPO_2_ in syrup group was not significantly different from one time to another. In contrast, significant (but within normal) changes in SPO_2_ were observed between minute 30 and other measurement times among EF group: *P* values: 0.015, 0.006, and 0.009 for baseline, minute 0, and minute 15, respectively (adjustment Bonferroni test).

#### 3.2.2. Heart Rate

Subjects in group A (syrup) had a higher baseline heart rate in comparison to group B. *P* < 0.01, and this pattern continued at all measurement times except minute 0. Intragroup measurements did not show any significant change from baseline to minute 30: repeated measure of Greenhouse *P* > 0.05.

#### 3.2.3. Respiratory Rate

Respiratory rate was higher basically and in all other measurements except minute baseline, in group A. There was a mild descending curve from baseline to minute 30 in this group: Mann-Whitney test, *P* = 0.004.

#### 3.2.4. Systolic and Diastolic Blood Pressure

The groups were not significantly different. Intragroup measurements revealed significant fluctuations in systolic and diastolic blood pressure in group B: repeated measure of Greenhouse *P* value: 0.02 and 0.001 for systolic and diastolic pressure, respectively.

Serious adverse effects were not observed during and after sedation appointment. One case of hiccupping was observed shortly after drug administration.

## 4. Discussion

The results of this study indicated that both regimens were safe with reasonable level of sedation acquired. To the best of our knowledge, there are few published studies that have investigated safety and efficacy of midazolam syrup versus orally administered IV midazolam (EF midazolam). In comparison to commercial syrup with IV midazolam in the mixture of Syrpalta (pH = 5), Brosius and Bannister reported higher plasma levels and superior sedative effects from IV midazolam-Syrpalta [13]. Khalil et al. reported less preanesthetic anxiety at 15 minutes and at parental separation in children who received IV midazolam-Syrpalta mixture when compared to those who received the premixed midazolam solution [14].

The effectiveness of EF midazolam over commercial preparations is a controversial issue [2, 5]. Our results did not show a significant difference between two midazolam preparations; however, administration of the commercial syrup increased the chance of overall success when compared to EF preparation. Variability of sweeteners and juices added to IV drug resulted in diversity in pH, viscosity, and pharmacokinetics that may explain this controversy [15–18]. The pH of our oral dosage was 3.7 and the pH of commercially syrup was 4 and fruit juices usually added to midazolam had a pH about 2.8 that can justify the difference in absorption of drug [15, 19]. Mucosal absorption is a pH dependent phenomenon. Increasing the pH from 2.8 (IV formulation) to 4 results in formation of more than 95% of active lipophilic drug or closed ring that is readily absorbable from oral mucosa [13, 14]. Since significant amount of midazolam is absorbed by mucosal membranes of the oral cavity, esophagus, and stomach, pH variability may result in divergent observed responses to midazolam [19]. Another consideration in midazolam absorption is the drug's viscosity. Viscous preparations are retained in oral environment for longer periods of time that* per se* increases the local absorption of drug from permeable oral mucosa especially nonkeratinized tissue of buccal mucosa [16, 18, 20, 21]. Relative absorption of drug from direct drainage of oral blood vessels into the jugular vein is another priority over less viscous preparations which lead to better bioavailability of drug [20].

In the present study a dose of 0.5 and 0.2 mg/kg was chosen for children under and over five years of age, respectively, according to a pilot study as well as the previous works on this area [3, 22]. Age effects on dosage requirements have been discussed in the anesthesiology literature [17]. The midazolam dose should be individualized based on patient age, degree of anxiety, and the level of sedation desired [3]. Younger children may need higher doses of the drug for higher hepatic blood flow and metabolism rate [16, 23], in addition to lower expression, distribution, and coupling ability of type A GABA receptors [17]. It appears to be an optimal balance between anxiolytic activity and side effect liability in doses of 0.25 mg to 0.50 mg/kg [3]. We compared all aspects of sedation including sleep, movement, crying, and overall behavior by adjusting the effect of age. Similar results were achieved when the total dose was restricted to decrease the dose in older children. Although midazolam falls in a group of agents with broad margin of safety, lowering the dose may be desirable for decreasing the potential side effects [3, 5]. It may be logical to preserve the higher doses for those who do not respond favorably to lower doses of drug.

Mild and moderate sedation via oral route is an effective, safe, and convenience mode of midazolam administration that may fill the gap between psychological strategies and general anesthesia in many subjects.

More research is needed either by increasing the prescribed dose of drug or by administration of alternative agents in cases of unsuccessful sedations.

## 5. Conclusion

According to the results of this study EF midazolam preparations in a dose of either 0.2 or 0.5 mg/kg provided safe and effective sedation. Hence, lower doses may be advocated to older children.

## Clinical Implications

- What is already known is that midazolam is widely used as a safe and effective sedative agent in pediatric sedation. However, oral form of drug is not available in many countries and it is also expensive and not cost effective for occasional use.
- What this article adds is that injection formulation can be used for oral administration.
- Implications for translation are as follows. This extemporaneous preparation may be effectively used by pediatric dentists and other professionals who work in pediatric area as a safe effective agent in management of uncooperative behavior. In addition, identifying the populations who benefit from this agent can help to target the responders. Further researches can focus on other doses of midazolam or alternative agents for children with those behavioral characteristics that are not responsive to midazolam.

## Figures and Tables

**Table 1:** Demographic characteristics of children.

	Syrup	EF	Significant level
Age (months)	72.4 ± 11.8	73.4 ± 8.2	*P* = 0.14^¶^
Age range	48–84	50–84	

Body weight (kg)	20.2 ± 5.5	20.5 ± 5.6	*P* = 0.77^¶^
Weight range	14–33	13–35	

Male/female (*n*)	19/25	22/22	*P* = 0.41^∗^

^¶^Independent test, ^∗^Chi-square.

**Table 2:** Frequency of exhibited behavior according to North Carolina (NC) behavior rating scale.

Evaluation time	Group	Quiet *n* (%)	Annoyed *n* (%)	Upset *n* (%)	Wild *n* (%)	*P* value^¶^
Child arrival	Syrup EF	0 (0) 0 (0)	27 (61.4) 28 (63.6)	14 (31.8) 10 (22.7)	3 (6.8) 6 (13.6)	*P* = 0.43

Injection	Syrup EF	17 (38.6) 21 (41.7)	20 (45.5) 17 (38.6)	7 (15.9) 6 (13.6)	0 (0) 1 (2.3)	*P* = 0.52

Cavity preparation	Syrup EF	24 (54.5) 19 (43.2)	14 (31.8) 16 (36.4)	6 (13.6) 7 (15.9)	0 (0) 2 (4.5)	*P* = 0.23

Total	88	43 (48.9)	31 (35.2)	12 (13.6)	2 (2.3)	

^¶^Friedman test.

**Table 3:** Definition of North Carolina rating scale [12].

Behavior	Definition
Quiet	Patient is quiet or sleeping with only extraneous, inconsequential movements

Annoyed	Patient is cooperative for treatment but with 1 or 2 undesirable behaviors

Upset	Patient noticeably disturbed, with 2 to 3 undesirable behaviors^∗^ present, making treatment difficult

Wild	Patient extremely defiant with presence of all undesirable behaviors^∗^ making treatment extremely difficult

^∗^Undesirable behavior includes crying, screaming, head movement, torso movement, and foot movement.
